# Transcriptomic biomarkers for predicting response to neoadjuvant treatment in oesophageal cancer

**DOI:** 10.1093/gastro/goaa065

**Published:** 2021-01-08

**Authors:** Anita Lavery, Richard C Turkington

**Affiliations:** Patrick G Johnston Centre for Cancer Research, Queen’s University Belfast, Belfast, UK

**Keywords:** oesophageal cancer, predictive biomarkers, chemotherapy, radiotherapy, gene expression, pathological response

## Abstract

Oesophageal cancer is a devastating disease with poor outcomes and is the sixth leading cause of cancer death worldwide. In the setting of resectable disease, there is clear evidence that neoadjuvant chemotherapy and chemoradiotherapy result in improved survival. Disappointingly, only 15%–30% of patients obtain a histopathological response to neoadjuvant therapy, often at the expense of significant toxicity. There are no predictive biomarkers in routine clinical use in this setting and the ability to stratify patients for treatment could dramatically improve outcomes. In this review, we aim to outline current progress in evaluating predictive transcriptomic biomarkers for neoadjuvant therapy in oesophageal cancer and discuss the challenges facing biomarker development in this setting. We place these issues in the wider context of recommendations for biomarker development and reporting. The majority of studies focus on messenger RNA (mRNA) and microRNA (miRNA) biomarkers. These studies report a range of different genes involved in a wide variety of pathways and biological processes, and this is explained to a large extent by the different platforms and analysis methods used. Many studies are also vastly underpowered so are not suitable for identifying a candidate biomarker. Multiple molecular subtypes of oesophageal cancer have been proposed, although little is known about how these relate to clinical outcomes. We anticipate that the accumulating wealth of genomic and transcriptomic data and clinical trial collaborations in the coming years will provide unique opportunities to stratify patients in this poor-prognosis disease and recommend that future biomarker development incorporates well-designed retrospective and prospective analyses.

## Introduction

Oesophageal cancer is a devastating disease with a poor prognosis and limited treatment options, and has been appropriately designated a ‘cancer of unmet need’ by Cancer Research UK [1]. Globally, oesophageal cancer is the seventh most common cancer and the sixth leading cause of cancer death, with a 5-year overall survival rate of 10%–30% in most countries worldwide [2, 3]. Histologically, the two most common subtypes are oesophageal adenocarcinoma (OAC) and oesophageal squamous-cell carcinoma (OSCC). These differ significantly in terms of incidence, geographical distribution, risk factors, and tumour biology [4–6]. OSCC accounts for 90% of oesophageal cancer worldwide and is the predominant subtype in South-East and Central Asia [7]. In Western countries, OAC predominates and, alarmingly, the rates of OAC have risen rapidly over the past 30 years in Western populations, with the highest incidence being in the UK and the Netherlands [4, 8, 9]. Genomic profiling has demonstrated that OAC and OSCC are biologically distinct. Analysis of DNA methylation, messenger RNA (mRNA) and microRNA (miRNA) expression, and somatic copy-number alterations in oesophageal cancer has demonstrated that OAC bears greater similarity to the chromosomally unstable molecular subtype of gastric cancer than OSCC, whereas OSCC more closely resembles squamous-cell head and neck carcinoma than OAC [5].

In the setting of resectable disease, there is clear evidence that neoadjuvant treatment in the form of neoadjuvant chemotherapy, perioperative chemotherapy, or neoadjuvant chemoradiotherapy results in improved survival [10–16]. There is currently insufficient high-quality evidence to support the superiority of one modality over another and results of comparative trials such as the Neo-AEGIS and ESOPEC trials in OAC and the NExT study in OSCC are awaited [17–19]. Specific chemotherapy regimens and preference for neoadjuvant chemotherapy or neoadjuvant chemoradiotherapy differ worldwide, with the main aims being to downstage the tumour, increase R0 resection rates, and eradicate micro-metastatic disease. Regarding perioperative chemotherapy, the most promising results come from the FLOT4-AIO trial, which included a subgroup of patients with gastro-oesophageal-junction carcinomas and reported 5-year overall survival rates of 36% with ECF/X (epirubicin and cisplatin plus 5-fluorouracil [5-FU]/capecitabine) and 45% with FLOT (5-FU, leucovorin, oxaliplatin, and docetaxel) [12].

The ability to downstage the tumour through gaining a pathological response is, however, limited. A pathological response to neoadjuvant chemotherapy and neoadjuvant chemoradiotherapy in oesophageal cancer, defined as complete resolution of tumour or the presence of only scattered tumour cells (tumour-regression grade [TRG] 1–2), is independently predictive of improved overall survival [20–23]. Disappointingly, only 15% of resected tumours demonstrate a histopathological response to neoadjuvant chemotherapy and 25%–30% to neoadjuvant chemoradiotherapy [24–26]. Importantly, neoadjuvant treatment also has a significant toxicity burden, with >50% of patients experiencing grade 3 or 4 toxicities in some trials, with potential resultant delays in time to surgical resection and missed opportunities to avail of clinical trial options [12, 16]. Given the relatively small chance of benefit, associated toxicity, and potential quality-of-life implications for patients, it is crucial that we prospectively identify responders prior to instituting treatment. In oesophageal cancer, this has proved challenging and there are currently no predictive biomarkers for response to neoadjuvant therapy in routine clinical use.

In oesophageal cancer, outcomes for patients with similar demographic characteristics and stage of disease following neoadjuvant therapy and surgery are highly variable. Clinical factors, including TNM stage and tumour location, are not reliable predictors of response to neoadjuvant therapy and it has been hypothesized that the differences in response rates could be due to alterations in tumour biology [24, 25]. Tumour biopsies are a rich source of information about tumour biology and potentially chemo- and radio-sensitivity. The rapid development of -omics technologies, in particular gene expression profiling, allows the comprehensive assessment of thousands of genes at one time and is a powerful technology with which to explore factors affecting treatment response. As genomic biomarkers have been widely reviewed previously, we will focus our attention in this review on transcriptomic biomarkers [27–29]. We will outline progress to date in evaluating predictive transcriptomic biomarkers for neoadjuvant therapy in oesophageal cancer and discuss the challenges facing biomarker development in this patient group.

## Approaches to cancer biomarker development and reporting

### Biomarker development

Broadly, cancer biomarkers are classified into two categories. A predictive biomarker is defined as ‘a biomarker used to identify individuals who are more likely than similar individuals without the biomarker to experience a favourable or unfavourable effect from exposure to a medical product or an environmental agent’ [30, 31]. Predictive biomarkers can be invaluable in choosing the optimal treatment course for a patient; for example, HER2 status predicts response to Trastuzumab in metastatic gastric cancer [32]. A prognostic biomarker relates to the risk of future clinical outcomes independently of treatment and is defined as ‘a biomarker used to identify likelihood of a clinical event, disease recurrence or progression in patients who have the disease or medical condition of interest’; for example, nodal status in oesophageal cancer [30, 31, 33, 34].

Biomarker development is a complex, expensive, and often lengthy process requiring high-quality studies with comprehensive validation. Structured guidelines, such as those from the Institute of Medicine, have been derived in an effort to standardize approaches and improve quality [35]. The development of a biomarker with clinical impact encompasses several key stages: biomarker discovery, assay development, analytical and clinical validation, and clinical utility [33].

Prospective randomized trials remain the optimal method for establishing clinical utility in biomarker development; however, this is not always feasible in terms of cost or sample size [36, 37]. If researchers wish to use archival tissue, Simon *et al.* recommend prospective–retrospective study designs in which archived samples from prospective clinical trials are used to assess a biomarker [38]. The assay is performed only after a biomarker-evaluation protocol has been written and assay evaluation is blinded to clinical data. Similarly, Pepe *et al*. recommend a prospective-specimen-collection retrospective-blinded-evaluation (PRoBe) design [36]. This nested case–control study design incorporates prospective sample collection prior to blinded outcome assessment and subsequent biomarker verification. Although the authors focus on biomarker-evaluation studies, the core facets of this design would strengthen many predictive biomarker studies.

### Reporting biomarker studies

The REporting recommendations for tumour MARKer prognostic studies (REMARK) framework represents an important benchmark standard for reporting biomarker studies [37, 39, 40]. The authors’ focus is on prognostic studies, but the principles are broadly translatable to predictive biomarker discovery and evaluation as the REMARK 20-point checklist incorporates key phases of biomarker development. Although the emphasis is on reporting rather than study design and conduct, adherence to these guidelines has the potential to improve both study quality and biomarker utility. Many key publishers have endorsed the REMARK framework; however, adherence is sporadic. With these guidelines and frameworks in mind, we will assess the predictive biomarker landscape of resectable oesophageal cancer.

## Predictive biomarkers in oesophageal cancer

Identifying and adequately validating a reliable predictive biomarker for response to therapy in oesophageal cancer has proved challenging to date and individualized, stratified treatment remains an unrealized goal in the neoadjuvant setting. In recent years, the expansion in technology for biomarker detection has presented new opportunities for robust biomarker development. A series of studies have sought to evaluate predictive genomic and transcriptomic biomarkers in oesophageal cancer. Broadly, these have evaluated single genes, mRNAs, and miRNAs as potential biomarkers. With regard to epigenetics, there are few reports relating to DNA methylation and chemosensitivity in oesophageal cancer [41, 42]. This review will primarily focus on the development of predictive transcriptomic biomarkers for predicting response to neoadjuvant treatment in oesophageal cancer.

### Single-gene biomarkers, gene panels, and epigenetics

To date, numerous single-gene predictive biomarkers have been studied, where mutational changes or changes in the expression of one individual gene are used to predict the response to neoadjuvant treatment and these studies have previously been extensively reviewed [28, 29, 43–45]. Reported biomarkers predictive of response include cell-cycle regulators (*CDC25B*, *Cyclin D1*), DNA-repair genes (*p53*, *ERCC1*), and genes involved in 5-FU metabolism. Regarding predictive gene panels assessing several gene mutations, we found no relevant studies in the neoadjuvant setting in oesophageal cancer on reviewing the literature, although predictive gene panels have been investigated in the setting of advanced disease. Okines *et al*. investigated the predictive impact of mutations in *KRAS*, *BRAF*, *PIK3CA*, and *PTEN* expression in a cohort of patients with inoperable oesophageal or gastric cancer from the REAL3 trial [46]. There was no relationship between mutational status and response-evaluation criteria in solid tumours (RECIST) response but low mutational frequency limited the power of this study. Despite numerous single-gene studies, none of these biomarkers has been brought forward to clinical use, paving the way for a new approach.

Only a small number of studies have examined the role of predictive epigenetic biomarkers in oesophageal cancer [41, 42]. In one genome-wide methylation analysis of 104 patients with OSCC, methylation of ZNF695, a zinc-finger protein, was independently predictive of chemoradiotherapy response [41]. The authors hypothesized that ZNF695, thought to be a transcription factor, regulated the expression of DNA-repair genes involved in repairing the DNA damage resulting from chemoradiotherapy. Chang *et al*. used genome-wide methylation analysis and pyrosequencing on pretreatment endoscopic OSCC biopsies to derive a risk score composed of a six-CpG panel of DNA methylation biomarkers (located in *KCNK4*, *IFNGR2*, *PAX6*, *NOTCH4*, *NPY*, and *SOX17*) predictive of poor chemoradiotherapy response with AUC 0.930 [42].

Given the molecular complexity and heterogeneous landscape of oesophageal cancer and the likelihood that multiple pathways contribute to the sensitivity or resistance to neoadjuvant treatment, single genes or limited panels of biomarkers may not adequately reflect the molecular landscape of a tumour. The vast majority of these studies have been performed in underpowered cohorts without adequate validation and so have not progressed to routine clinical use. Considering the small number of studies and low clinical impact of this set of biomarkers, we will instead focus on the development of transcriptomic biomarkers that are predictive of response to therapy and not simply prognostic.

### Transcriptomic biomarkers

Gene expression profiling is a powerful tool that is now increasingly being used in cancer screening, diagnostics, prediction, and treatment planning. The most widely used methods are microarray analysis and RNA sequencing (RNA-seq), although reverse transcription–polymerase chain reaction (RT–PCR) assays may also be employed. Transcriptomic biomarkers are particularly complex, as they are generated using high-dimensional data and sophisticated computational modelling. This introduces major challenges, as rigorous statistical, bioinformatics, laboratory, and clinical procedures are required to develop and validate these tests and evaluate their clinical utility.

#### mRNA biomarkers

A total of 16 studies describe mRNA biomarkers predicting response to neoadjuvant therapy prior to resection (Table 1); however, considerable heterogeneity exists between the studies in both design and outcome measures (Supplementary Table 1). Biomarker analysis was performed on pretreatment biopsy samples, using fresh frozen tissue samples in the primary data set in all but three of the studies. Turkington *et al*. validated a 44-gene assay previously developed in breast cancer, the DNA damage immune response (DDIR) assay, in routine clinical formalin-fixed paraffin-embedded (FFPE) biopsies increasing the clinical applicability of this biomarker [47]. McLaren *et al*. also used FFPE endoscopic biopsies to evaluate the expression of 11 genes using RT–PCR [49]. Most treatment regimens involved platinum agents and 5-FU, although exact regimens varied significantly and the majority of studies assessed pathological response but utilized a variety of classification systems and cut-offs. For example, Turkington *et al*. used the Mandard classification (response: TRG 1–2, fibrosis with no tumour or scattered tumour cells), whereas Schauer *et al*. utilized the Becker tumour-regression grading system (response: <50% viable tumour cells) [21, 47, 51, 63]. Two studies assessed radiological response only with various imaging modalities employed, including computed tomography (CT) and endoscopic ultrasound (EUS) [50, 55]. The studies listed predominantly utilized microarrays and 10 different array platforms were employed across 16 studies. Finally, pathological subtype (OAC, OSCC, or mixed) varied between studies and this heterogeneity of samples and methodology poses challenges when interpreting study findings.

**Table 1.** Studies reporting mRNAs associated with response to neoadjuvant therapy in OAC and OSCC

Author (country)	Year	Platform	No. of patients	Pathology (cases)	Sample type	Neoadjuvant treatment	Response assessment	Responder definition	Signature	Validation (cases)
Turkington (UK) [47]	2019	Almac Xcel array DDIR assay (44 genes)	273	OAC	FFPE	(Epirubicin)/Cisplatin/5-FU or Capecitabine; Oxaliplatin/Capecitabine NACT	Resection	TRG1 or TRG2	DDIR signature (44 genes)	44 genes (273)
MacGregor (UK) [48]	2018	Illumina HumanHT-12-v3 Expression BeadChips	38	OAC	Fresh frozen or FFPE	Oxaliplatin/5-FU NACT	Resection	TRG1–TRG3	7 DNA-repair genes	Not performed^a^
McLaren (USA) [49]	2017	qPCR for 11 genes	29	OAC	FFPE	Carboplatin/paclitaxel/(5-FU); Cisplatin/5-FU NACRT	Resection	No residual tumour cells	2 genes: *CCL28* and *DKK3*	Not performed
Rao (UK) [50]	2011	CRUK DMF 22K v1.0.0 cDNA microarray	35	OAC	Fresh frozen	Epirubicin/Cisplatin/Capecitabine NACT	Radiological (CT and EUS)	RECIST criteria	113 genes	LOOCV
Schauer (Germany) [51]	2010	Affymetrix U133 Plus 2.0 microarray	47	OAC	Fresh frozen	Cisplatin/5FU/Leucovorin NACT	Resection	<50% residual tumour cells	86 genes	Not performed
Luthra (USA) [52]	2007	Affymetrix U133A Gene Chip	19	OAC	Fresh frozen	Docetaxel/5-FU/Irinotecan NACRT	Resection	No residual tumour cells	IVL	Not performed
Fujishima (Japan) [53]	2017	Agilent SurePrint G3 Human Gene Expression v3 8x60K microarray	32	OSCC	Fresh frozen	Docetaxel/Cisplatin/5-FU NACT	Resection	No residual tumour cells	17 molecules	12 molecules (7)
Wen (China) [54]	2014	Affymetrix U133 Plus 2.0 microarray	28	OSCC	Training: pooled fresh frozen Validation: FFPE	Cisplatin/Vinorelbine NACRT	Resection	No residual tumour cells	3 genes: *LIMCH1*, *MMP1*, and *C1orf226*	3 genes: *LIMCH1*, *MMP1*, and *C1orf226* (32)
Motoori (Japan) [55]	2010	AceGene 30K oligonucleotide microarray	25	OSCC	Fresh frozen	Cisplatin/5-FU/Doxorubicin NACT	Radiological (CT)	>50% tumour reduction	199 genes	199 genes (10)
Pühringer-Oppermann (Germany) [56]	2010	RT–PCR (3 genes)	97	OSCC	FFPE	Cisplatin/5-FU; Oxaliplatin/5-FU; 5-FU NACRT	CT Endoscopy Resection	CR/PR <10% residual tumour cells	SMAD4	Not performed
Bollschweiler (Germany) [57]	2016	TaqMan low-density arrays	85	OAC (56) OSCC (29)	Fresh frozen	Cisplatin/5-FU NACRT	Resection	<10% residual tumour cells	3 genes: *ERCC1*, *DPYD*, and *ERBB2* + ERCC1-SNP	Not performed
Warnecke-Eberz (Germany) [58]	2010	TaqMan low-density arrays	41	OAC (17) OSCC (24)	Fresh frozen	Cisplatin/5-FU NACRT	Resection	<10% residual tumour cells	17 genes (ANN analysis)	Not performed
Metzger (Germany) [59]	2010	ABI Human Genome Survey 2.0 microarray	66	OAC (26) OSCC (40)	Fresh frozen	Cisplatin/5-FU NACRT	Resection	<10% residual tumour cells	OAC: CUL2 + STK11 OSCC: CUL2	Not performed
Maher (Ireland) [60]	2009	ABI Human Genome Survey microarray	13	OAC (10) OSCC (3)	Fresh frozen	Cisplatin/5-FU NACRT	Resection	TRG1 or TRG2	12 genes	8 of 12 genes (27)
Duong (Australia) [61]	2007	Peter Mac 10.5K cDNA microarray	46	OAC (25) OSCC (21)	Fresh frozen	Cisplatin/5-FU NACRT	Radiological (CT, FDG-PET) Repeat biopsy Resection	Complete response No residual tumour cells	32 genes (OSCC only)	LOOCV
Luthra (USA) [62]	2006	Affymetrix U133A Gene Chip	19	OAC (16) OSCC (2) ASCC (1)	Fresh frozen	Docetaxel/5-FU/Irinotecan NACRT	Resection	No residual tumour cells	3 genes: *PERP*, *S100A2*, and *SPRR3*	Not performed

^a^ Validation performed using another group of genes.

5-FU, 5-fluorouracil; ANN, artificial neuronal network; ASCC, adenosquamous carcinoma; CR, complete response; DDIR, DNA-damage immune response; EUS, endoscopic ultrasound; FFPE, formalin-fixed, paraffin-embedded; LOOCV, leave-one-out cross-validation; NACT, neoadjuvant chemotherapy; NACRT, neoadjuvant chemoradiotherapy; OAC, oesophageal adenocarcinoma; OSCC, oesophageal squamous-cell carcinoma; qPCR, quantitative polymerase chain reaction; PR, partial response; RECIST, Response Evaluation Criteria in Solid Tumours; RT–PCR, reverse transcription–polymerase chain reaction; TRG, tumour-regression grade.

Microarrays allow a comprehensive assessment of thousands of transcripts simultaneously but have several significant limitations [64]. First, a microarray can only assess gene expression for a set of pre-specified probes, potentially limiting the chance of novel findings. Second, high background levels due to cross-hybridization can reduce the accuracy of gene expression results [65, 66]. Finally, differences in data normalization (performed to account for differences in hybridization, labelling, and detection methodology) and in filtering cause significant variation in results and caution must be exercised when comparing results from different assays and laboratories [64, 67, 68].

High-throughput RNA-seq has revolutionized transcriptome profiling and has many advantages, including low background signal, a large dynamic range of detection, and excellent reproducibility, and is not limited by the detection of pre-specified transcripts [69]. However, it is significantly more expensive, requires a higher quality of input material, and requires high-powered computing support with complex bioinformatic analysis methods.

#### Oesophageal adenocarcinoma

Six studies explored OAC alone and varied significantly, reporting genes involved in a wide variety of biological pathways [47–52]. The studies used different analysis platforms, including commercially available and in-house microarrays, and different treatment regimens, reflecting the geographical variation in neoadjuvant therapy. In one OAC study, out of the 86 genes significantly differentially expressed between neoadjuvant chemotherapy responders compared with non-responders, the most common were tumour-suppressor genes, tyrosine kinase receptors, and those involved in apoptosis, cell–cell interactions, and the cytoskeleton [51]. Upregulation of *Ephrin B3*, which is regulated by the Wnt pathway and known to be involved in chemosensitivity, showed the strongest association with response. Another pathway—the *TP53*-dependent apoptosis pathway—was implicated in affecting OAC chemosensitivity by Rao *et al*. [50]. Among the 113 differentially expressed genes in this study, *PERP* is an effector in this pathway and has previously been implicated in chemoradiotherapy response in OAC and OSCC [50, 55, 62, 70]. These examples indicate the diversity of findings in mRNA OAC studies.

McLaren *et al*. preselected 11 candidate genes based on their association with oesophageal cancer prognosis and found that overexpression of *CCL28* and underexpression of *DKK3* were significantly associated with pathological complete response to neoadjuvant chemoradiotherapy [49]. *CCL28* recruits T regulatory cells, is involved in regulation of the immune response, and was previously identified as being associated with chemotherapy response by Maher *et al*.; however, in that case, *CCL28* was downregulated [60, 71–73]. As part of a prospective translational clinical trial, MacGregor *et al*. analysed a panel of 280 DNA-repair genes [48]. Relative overexpression of seven DNA-repair genes was significantly associated with lack of pathological response to oxaliplatin-based neoadjuvant chemotherapy. Using immunohistochemistry (IHC) to further evaluate candidate biomarkers, low levels of XPF (closely related to *ERCC1*) were associated with treatment response, but no association was found between XPF and prognosis in a matched cohort of patients treated with surgery alone, indicating that this is a biomarker of response to chemotherapy.

Turkington *et al*. reported a gene expression signature with predictive and prognostic effect in a cohort of 273 OAC patients [47]. The 44-gene DDIR assay (Almac Diagnostics, Craigavon, Northern Ireland) was applied to gene expression microarray data from pre-chemotherapy OAC samples. In this retrospective analysis, DDIR-positive patients had significantly improved relapse-free and overall survival, and the DDIR assay was independently predictive for response to neoadjuvant DNA-damaging chemotherapy in OAC. Notably, DDIR positivity was associated with a pro-inflammatory, ‘immune-hot’ biology with elevated levels of PDL-1 and CD8 T lymphocytes. To fully ascertain the clinical utility of the DDIR signature, further retrospective validation in a randomized clinical trial data set followed by prospective validation is required. In summary, each of these studies reports a range of different genes involved in a wide variety of pathways and biological processes, and this is explained to a large extent by the different platforms and analysis methods used. The majority of studies are also underpowered and so not suitable for identifying candidate biomarkers.

#### Oesophageal squamous-cell carcinoma

Four studies examined mRNA biomarkers in OSCC alone and, similarly to OAC, they varied significantly [53–56]. Motoori *et al*. derived and validated a 199-gene signature predictive of radiological (by CT) response to neoadjuvant chemotherapy with 82% accuracy [55]. Also in OSCC, Wen *et al*. derived a three-gene model (*LIMCH1*, *MMP1*, *C1orf226*) that was predictive of pathological complete response to neoadjuvant chemoradiotherapy [54]. None of the 10 most differentially expressed genes in this study showed an overlap with previous reports regarding neoadjuvant chemoradiotherapy in oesophageal cancer and the authors cited the use of different patient groups and microarray platforms as a possible explanation. Fujishima *et al*. used microarray data and molecular expression analysis to identify 17 molecules as predictors of pathological complete response, which were associated with pathways including transcriptional regulation by *STAT*, *SMAD*, and *RB/E2F* [53]. In a separate study focusing on the TGF-β pathway, known to be involved in cell-cycle arrest and apoptotic cell death, increased *SMAD4* expression was significantly higher in tumours with total or partial regression compared to those with little or no regression [56, 74]. The authors hypothesized that *SMAD4* could be a rate-limiting step in the establishment of cell-cycle arrest and apoptosis in OSCC.

#### Mixed oesophageal adenocarcinoma and squamous-cell carcinoma

Several studies combined the analysis of both OAC and OSCC, and, similarly to the reports previously discussed, implicated a range of genes and pathways in therapy response [57–62]. One of the earliest gene expression profiling studies in oesophageal cancer utilized unsupervised hierarchical clustering to derive a three-gene combination (*PERP*, *SPPR3*, and *S100A2*), overexpression of which successfully discriminated between pathological complete response and non-response to neoadjuvant chemoradiotherapy with sensitivity and specificity of 85% [62]. Two of these genes (*S100A2* and *SPRR3*) are located at the epidermal differentiation complex and the same group subsequently showed that downregulation of gene expression in this region was associated with resistance to neoadjuvant chemoradiotherapy, albeit in a small cohort of 19 patients [52]. Focusing on a different pathway, Warnecke-Eberz *et al*. showed that reduced expression of *DPYD*, a rate-limiting enzyme in 5-FU metabolism, was independently associated with histopathological response to neoadjuvant chemoradiotherapy, indicating the importance of this enzyme in 5-FU sensitivity [58]. Further artificial neuronal network analysis using a 17-gene model predicted sensitivity to cisplatin/5-FU neoadjuvant chemoradiotherapy with 85% accuracy. In a subsequent prospective study by the same group in OAC and OSCC patients, *ERCC1* rs11615 single nucleotide polymorphism (ERCC1-SNP) combined with expression of *ERCC1*, *DPYD*, and *ERBB2* was predictive of a minor pathological response to neoadjuvant chemoradiotherapy with an accuracy of 80% [57]. Maher *et al*. developed a five-gene model (*EPB41L3*, *RNPC1*, *RTKN*, *STAT5B*, and *NMES1*) that predicted pathological response to neoadjuvant chemoradiotherapy and was one of the few groups to test the performance of the biomarker in an independent validation cohort [60]. Overall, as with publications examining OAC and OSCC alone, there is wide variation between these studies, reflective of the broader issues in biomarker discovery in general.

Importantly, publications including both OAC and OSCC reported varying results depending on the pathological subtype. In a mixed group of OAC and OSCC patients treated with neoadjuvant chemoradiotherapy, Duong *et al*. derived a 32-gene classifier that correctly identified 10 out of 15 non-pathological complete responses in OSCC. The classifier was not predictive in OAC [61]. Similarly, Metzger *et al*. reported that increased mRNA expression of *CUL2*, involved in cell-cycle progression, was predictive of major histopathological response in OAC and OSCC, whereas increased expression of *STK11*, a tumour suppressor, was predictive in OAC only [59]. The differences between OAC and OSCC are reflective of the known distinct biology of each subtype and are illustrative of the potential pitfalls of combining both subtypes in biomarker studies.

#### mRNA biomarkers and the Hallmarks of Cancer

A wide range of genes with diverse functions were associated with response to neoadjuvant treatment in mRNA studies. In order to further understand the biology associated with response, we categorized each of the predictive genes reported in Table 1 according to the most recently updated Hallmarks of Cancer [75]. The categorization was performed initially by matching the Gene Ontology terms associated with each gene with the relevant hallmark according to the categorization table previously published by Knijnenburg *et al*. [76, 77]. For those genes for which no hallmark was derived using this method, hallmarks were assigned according to gene function and key involved pathways using the Kyoto Encyclopaedia of Genes and Genomes and National Center for Biotechnology Information gene resource [78, 79]. Some genes were assigned to more than one hallmark.

**Figure 1.**
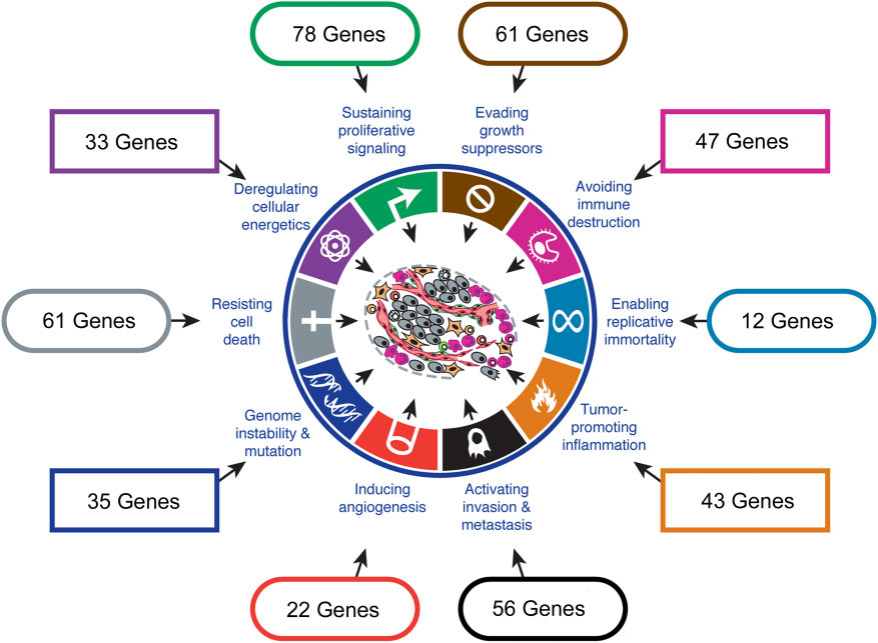
Genes associated with predicting response to neoadjuvant treatment in mRNA studies, categorized according to the Hallmarks of Cancer. Reproduced and modified with permission [75].

Figure 1 illustrates the number of genes linked to treatment response associated with each hallmark. The most frequently associated hallmarks were sustaining proliferative signalling, resisting cell death, and evading growth suppressors (Supplementary Table 2). The diverse biologies represented here are illustrative of the complexity in determining mediators of treatment response in oesophageal cancer and the significant challenge faced in deriving suitable predictive biomarkers.

#### microRNA biomarkers

miRNAs are small non-coding RNAs ∼20–22 nucleotides long that act as negative regulators of gene expression post-transcriptionally by sequence-specific binding to the 3' untranslated regions (UTR) of mRNAs [80–83]. miRNAs play a role in regulating the major cell processes including development, apoptosis, cell proliferation, cell migration, and metastasis [81, 84]. miRNAs have multiple targets, can affect key processes in cancer development, and can act as tumour suppressors or oncogenes [83].miRNAs are attractive as potential biomarkers; they are smaller and more stable than mRNAs and can be relatively easily extracted from plasma or serum, FFPE, and fixed frozen samples, in addition to a range of body fluids [85–89]. Significant interest has therefore been generated in the potential for miRNAs as diagnostic, prognostic, and predictive biomarkers in oesophageal cancer. Several studies have evaluated miRNAs as predictive biomarkers for response to neoadjuvant therapy in oesophageal cancer (Table 2 and Supplementary Table 3). A vast range of miRNAs have been postulated as biomarkers with little concordance between studies and the majority of reports utilize microarrays in combination with quantitative RT–PCR.

**Table 2.** Studies reporting miRNAs associated with response to neoadjuvant therapy in OAC and OSCC

Author (country)	Year	Platform	No. of patients	No. of miRNAs	Pathology (cases)	Sample type	Neoadjuvant treatment	Response assessment	Responder definition	Biomarker	Validation (cases)
Chiam (Australia) [90]	2018	Illumina HiSeq 2500	31	283	OAC	FFPE	Cisplatin/5-FU NACRT	Resection	No residual tumour cells	miR-4521/miR-340-5p	miR-4521/miR-340-5p
										miR101-3p/miR-451a	miR101-3p/miR-451a
										miR-143-3p/miR-451a	miR- 143-3p/miR-451a (LOOCV)
Lynam-Lennon (Ireland) [91]	2016	Exiqon miRCURY LNA™Universal RT miRNA array	18	742	OAC	Fresh frozen	Cisplatin/5-FU NACRT	Resection	TRG1 or TRG2	miR-187	Not performed
Bibby (Ireland) [92]	2015	Exiqon miRCURY LNA™Universal RT miRNA array	18	742	OAC	Fresh frozen	Cisplatin/5-FU NACRT	Resection	TRG1 or TRG2	miR-330-5p	Not performed
Skinner (USA) [93]	2014	TaqMan MicroRNA Array	53	754	OAC	–	Cisplatin/5-FU; Oxaliplatin/5-FU NACRT	Resection	No residual tumour cells	miR-99b	miR-99b
		Fluidigm 48.48 Dynamic Array								miR-145*	miR-145*
		Illumina MicroRNA expression BeadChip								miR-451	miR-451
										miR-505*	miR-505*
											(65)
Slotta-Huspenina (Switzerland) [94]	2018	Agilent miRNA microarray	31	1,205	OSCC	FFPE	Cisplatin/5-FU; Oxaliplatin/5-FU NACRT	Resection	No residual tumour cells	miR-194*	Not performed
										miR-665	
Wen (China) [95]	2016	Agilent miRNA microarray	27	1,887	OSCC	Training: fresh frozen Validation: FFPE	Cisplatin/Vinorelbine NACRT	Resection	≤50% residual tumour cells	miR-145-5p	miR-145-5p
										miR-152	miR-152
										miR-193b-3p	miR-193b-3p
										miR-376a-3p	miR-376a-3p
											(79)
Sugimura (Japan) [96]	2012	TaqMan MicroRNA Array	74	365	OSCC	–	Cisplatin/Adriamycin/5-FU NACT	Resection + radiological (CT)	<2/3 residual tumour cells CT: PR/CR	let-7b	let-7b
										let-7c	let-7c
											(24)
Odenthal (Germany) [97]	2013	TaqMan MicroRNA Array	88	768	OAC (48) OSCC (40)	FFPE	Cisplatin/5-FU NACRT	Resection	<10% residual tumour cells	miR-192	Not performed
										miR-194	
										(OSCC)	
Ko (Canada) [98]	2012	Illumina miRNA BeadChip microarray	25	1,536	OAC (20) OSCC (5)	FFPE	Cisplatin/ Irinotecan NACRT	Resection	No residual tumour cells	miR-296	Not performed
										miR-141	
										miR-31	
										HS-240	
										HS_217	
Lynam-Lennon (Ireland) [99]	2012	Exiqon miRCURY LNA™Universal RT miRNA array	19	1	OAC (18) OSCC (1)	Fresh frozen	Cisplatin/5-FU NACRT	Resection	TRG1 or TRG2	miR-31	miR-31 (37)^a^

^a^ qRT–PCR validation in a cohort that included 19 patients from the initial-discovery cohort.

5-FU, 5-fluorouracil; CR, complete response; FFPE, formalin-fixed, paraffin-embedded; LOOCV, leave-one-out cross-validation; miRNA, microRNA; NACT, neoadjuvant chemotherapy; NACRT, neoadjuvant chemoradiotherapy; OAC, oesophageal adenocarcinoma; OSCC, oesophageal squamous-cell carcinoma; PR, partial response; TRG, tumour-regression grade.

#### Oesophageal adenocarcinoma

Following a similar theme to the mRNA studies, miRNA studies in OAC demonstrate heterogeneity in assay platforms and results, with few miRNAs being reported as significantly associated with response in more than one study. Skinner *et al*. were the first to propose a miRNA signature validated across several assay platforms to predict neoadjuvant chemoradiotherapy response [93]. A four-miRNA panel (miR-99b, miR-145*, miR-451, and miR-505*) was significantly predictive of pathological complete response in OAC patients treated with neoadjuvant chemoradiotherapy across discovery, model, and validation cohorts, each using a different analysis platform. The authors emphasized that the ability to remain predictive across three different assay platforms increased applicability. Using an alternative approach, Chiam *et al*. employed small-RNA sequencing to assess how the miRNA ratio (rather than specific levels) predicted response to neoadjuvant chemoradiotherapy in OAC [90]. Three ratios (miR-4521/miR-340-5p, miR101-3p/miR-451a, and miRNA 143-3p/miR-451a) had good cross-validated sensitivities and specificities for predicting pathological complete response.

Given its role in other malignancies, Lynam-Lennon *et al*. investigated the pretreatment expression of miR-187 in OAC in relation to neoadjuvant-chemoradiotherapy response. Levels were significantly reduced in poor responders with miR-187 also confirmed as having a functional role in sensitivity to cisplatin and radiotherapy *in vitro* [91]. In addition, DNA-damage-response genes (*NUPR1*, *SP100*, and *IFI16*) were downregulated following the overexpression of miR-187. Taken together, this suggests that miR-187 is involved in the regulation of pathways related to DNA damage and so response to platinum-based chemotherapy or radiotherapy. In a separate study, the same group also reported downregulation of miR-330-5p in non-responders [92]. Overall, the OAC studies used several platforms and reported a wide range of miRNAs involved in a variety of biological processes. Studies evaluating reproducibility across gene expression platforms have shown diverse results and this may account at least in part for the variation in the study results discussed here [67, 68, 100–102]. The small numbers involved in most studies reduce statistical power and this, together with the differences in patient characteristics and study methodology, may explain why none of these biomarkers has been brought forward to routine clinical use.

#### Oesophageal squamous-cell carcinoma

In OSCC, Sugimura *et al*. reported that let-7c played a role in chemosensitivity through regulation of the IL6/STAT3 pathway; low expression of let-7b and let-7c was associated with poor response to chemotherapy and low expression of let-7c was correlated with poorer overall survival [96]. Accordingly, upregulation of let-7c in OSCC cell lines increased sensitivity to cisplatin.

Two studies evaluated predictive biomarkers in OSCC using an Agilent microarray platform [94, 95]. Wen *et al*. assessed miRNA prediction of pathological response in patients receiving neoadjuvant chemoradiotherapy [95]. Their resultant support vector machine (SVM) model incorporating four miRNAs had accuracies of 100% and 87.3% in training and validation sets, respectively, for distinguishing pathological responders and non-responders. The SVM model was the only independent variable significantly associated with response to neoadjuvant chemoradiotherapy using multivariate analysis. An important strength of this study is the external validation of patients treated with the same neoadjuvant chemoradiotherapy regimen. Slotta-Huspenina *et al*. reported two miRNAs associated with response, miR-194* and miR-665, neither of which had been associated with response in previous studies [94]. Notably, patients with partial pathological regression were excluded from the analysis and the authors cited a small sample size and uncertainty regarding the prognostic significance of partial regression as mitigating factors. Although both these studies employed an Agilent experimental platform, they generated diverse biomarkers. This is often due to variation in patient characteristics, sample handling, and bioinformatic analyses employed in each study.

#### Mixed oesophageal adenocarcinoma and squamous-cell carcinoma

Studies combining miRNA profiling in both OAC and OSCC implicate a range of miRNAs in response to neoadjuvant treatment [97–99]. In a cohort of 88 patients, distinct miRNA profiles were seen for OAC and OSCC, and increased expression of two miRNAs (miR-192 and miR-194) was predictive of therapy response in OSCC but not OAC [97]. Ko *et al*. reported five different miRNAs (HS-240, miR-296, miR-141, miR-31, and HS_217) differentially expressed between pathological complete responders and non-responders but acknowledged the limitations of a small sample size (*n* = 25) [98]. Using quantitative PCR, Lynam-Lennon *et al*. analysed the expression of one of these, miR-31, in a combined cohort of OAC and OSCC patients receiving neoadjuvant chemoradiotherapy [99]. Reduced miR-31 expression was significantly associated with a poor pathological response. In line with this, they observed increased levels of miR-31-regulated DNA-repair genes. The authors postulated a possible chemoradiotherapy-resistance mechanism in which miR-31 alters the levels of DNA-repair genes in those exhibiting a poor response. The variation in virtually all aspects of these studies highlights the need for caution when incorporating mixed pathologies in predictive studies.

#### Long non-coding RNA biomarkers

Long non-coding RNAs (lncRNAs) are RNA molecules with >200 nucleotides that have little or no capacity for protein coding and have been shown to play important roles in the development and progression of oesophageal cancer [103–108]. There are two studies regarding the utility of lncRNAs in predicting response to neoadjuvant treatment in oesophageal cancer. Tong *et al*. showed that low expression of LOC285194, previously linked with poor outcomes in other cancer types, was the only independent risk factor associated with reduced response rates to neoadjuvant chemoradiotherapy in OSCC [109]. Low expression of LOC285194 was independently associated with significantly worse disease-free survival and overall survival. In OSCC patients, Chang *et al*. reported that expression of TUSC7, thought to act as a tumour suppressor, was upregulated in patients with a good radiological response to neoadjuvant chemotherapy compared with non-responders [110].

## Discussion

The goal of predictive biomarker research is to identify the treatment that results in the best outcome for each specific tumour biology. The gene expression biomarker studies outlined in this review aim to characterize a particular subgroup of tumours that respond to neoadjuvant therapy. Significant challenges still exist with interpreting the highly varied results, particularly in the context of the marked heterogeneity between studies and understanding the reasons behind response and non-response at the biological level.

To date, there are no validated genomic or transcriptomic biomarkers in clinical use in the neoadjuvant setting in oesophageal cancer. This ‘gap’ is reflective of broader issues in cancer biomarker development in which it is estimated that <0.1% of clinical biomarkers are translated from basic initial discovery studies to clinical use [111]. A proportion of these fail at the analytical and clinical validation stages; however, many are described only in the literature and are never brought from discovery to clinical validation [33]. Many common pitfalls at the discovery stage are relevant to the studies reviewed here.

The studies vary significantly in terms of participant characteristics, pathological subtype, type of sample used, treatment regimen, assay choice, statistical analysis, and whether validation is included. This variation, along with small sample sizes, may account for the lack of congruence between studies. Interpreting results in the context of such heterogeneity is challenging. Many of the studies outlined are retrospective in nature, which inherently introduces multiple sources of potential bias, such as selection bias and confounding factors. To reduce the risk of bias, blinding to clinical outcomes is widely recommended when conducting marker assessment [36, 39]. Taking into account the complexity of predictive biomarker studies, the REMARK authors clearly state that these studies should ideally occur in the context of prospective randomized trials in which the Consolidated Standards of Reporting Trials (CONSORT) guidelines apply [37, 112]. Taken together, these issues highlight the need for larger studies with more standardized approaches as well as the importance of reporting standards in biomarker development.

Biomarker studies to date have been hampered by small patient numbers and authors frequently cited the inadequate powering of their studies as a fundamental limitation [49, 52, 61, 90]. Incorporating small sample sizes relative to the number of gene expression measurements means that gene expression studies are vulnerable to overfitting, often due to failure to correct for multiple hypothesis testing [35]. Overfitting can occur when a computational modelling process unintentionally takes account of noise or other chance variables in a training data set so that genes that are predictive in the training data set are not predictive in a test data set. This highlights the importance of creating and maintaining high-quality, well-annotated specimen repositories, such as that maintained by the Oesophageal Cancer Clinical and Molecular Stratification (OCCAMS) Consortium [113]. An important strength of multi-institution biomarker studies with well-designed protocols is the potential for wider generalizability. One example of this is the high-quality retrospective analysis of randomized phase three clinical trial data sets, which led to the routine clinical use of KRAS as a biomarker for response to anti-EGFR therapies in colorectal cancer [114, 115]. Only by robustly testing and validating a biomarker in sufficiently powered cohorts can we generate meaningful outcomes and biomarkers to take forward into clinical practice.

In addition to appropriate statistical power, the correct choice of endpoint is critical in biomarker studies. It is important that endpoints reflect meaningful outcomes and those that matter to patients. The studies outlined used a range of classification systems to define pathological response, as previously described, and two used radiological response alone [50, 55]. CT response is notoriously difficult to discern in oesophageal cancer [116]. Furthermore, imaging using positron emission tomography (PET)–CT, CT, and EUS alone is not sensitive enough to detect pathological complete response [117]. The choice of endpoint should be carefully considered when evaluating potential biomarkers.

Validation is a key aspect of biomarker discovery and can occur within a data set or, ideally, using an independent sample set. Regarding initial validation, several studies reviewed here included training and test sets [61, 93, 96]. Others utilized cross-validation approaches in a single data set; this approach can be helpful if seeking to evaluate a marker combination, particularly where sample sizes are small [36, 50, 61, 90]. Seven studies used independent data sets for validation [53–55, 60, 93, 95, 96]. Ultimately, the highest level of evidence of validity comes from using an independent sample set that is not used to generate the initial computational model and this is widely recommended [36, 37]. Validation in independent data sets should be widely adopted in oesophageal cancer biomarker studies going forward.

In summary, the studies reviewed illustrate the many challenges involved in biomarker development in oesophageal cancer. Sample size, choice of endpoint, variation in treatment and analysis platform, and validation are recurring themes. Future studies should aim to address these using the framework provided by the REMARK criteria and integrate the results with information about tumour biology and molecular subtype.

A further consideration regarding predictive biomarkers in OAC is the insights they provide into the biological subtypes underlying response to therapy. Genomic and transcriptomic biomarkers differ from many other biomarkers in that the biological rationale and molecular mechanisms behind their predictive value are often unclear initially. The wide variation in study results demonstrates that the biological factors influencing response to neoadjuvant treatment in oesophageal cancer are not yet fully understood. It is clear, however, that a subgroup of responders exists and it may be that this represents a particular molecular subgroup yet to be fully defined. Recent molecular profiling studies have proposed molecular subtypes within the broad OAC and OSCC groups; however, it is unclear how many of these subgroups relate to clinical outcomes, including treatment response, and how the subgroups relate to each other [5, 118].

Several attempts to molecularly subtype OAC and OSCC and relate these groupings to treatment response and prognosis have been performed. The Cancer Genome Atlas conducted comprehensive multi-omics profiling of 164 oesophageal carcinomas revealing a strong molecular distinction between OAC and OSCC [5]. Secrier *et al*., as part of the UK OCCAMS Consortium, used a whole-genome sequencing-based approach to identify molecular subgroups in 129 OAC samples using pre-specified mutational signatures, previously described by Alexandrov *et al*. [118, 119]. This separated OAC into C > A/T-dominant, DNA-damage-repair impaired, and mutagenic subgroups. Importantly, no significant difference between any of the three groups could be found in terms of tumour grade or stage, response to chemotherapy, overall survival, recurrence-free survival, smoking, age, or sex. This may be due to the heterogeneous nature of the treatments applied to the cohort and the fact that mutational signatures, although representative of the mutational history of a tumour, are not necessarily representative of the current biology of a tumour. Similarly, of the 65 driver genes in OAC recently identified by Frankell *et al*., none was related to treatment response, with only *SMAD4* and *GATA4* mutations independently predicting reduced overall survival [120]. As previously outlined, using a 44-gene expression signature, the DDIR assay, we have identified a DNA-repair-deficient OAC subgroup with pro-inflammatory/immune biology. DDIR positivity was associated with improved response to neoadjuvant chemotherapy and significantly improved overall survival [47]. Whilst it is clear that a subgroup of clinically responding patients exists, the precise biology underpinning this phenotype remains elusive. The cellular response to DNA damage and its interplay with immune signalling may identify a subset of tumours primed for response to DNA-damaging chemotherapy or radiotherapy. However, further validation of existing biomarkers is required alongside the development and exploitation of rigorously collated, well-annotated, and sufficiently powered cohorts treated with relevant neoadjuvant therapy.

The future of predictive biomarker development in oesophageal cancer may also lie in the use of artificial intelligence, for example, through machine-learning techniques. These techniques have been used to predict treatment response in oesophageal cancer using imaging and clinicopathological data, alone and in combination [121, 122]. A recent study found that the use of deep neural networks, which capture key biological pathways related to treatment response, outperformed current machine-learning algorithms in predicting drug response [123]. The authors trained deep neural network models on a database of 1,001 cancer-cell lines and applied these models in a range of clinical cohorts, including the OAC OCCAMS data set [113]. The resultant model was able to successfully recognize biological pathways related to drug response. A key limiting factor is that oesophageal cancer is characterized by a high level of genomic instability and significant intra-tumoural heterogeneity [118, 124, 125]. In this setting, a precision medicine biomarker-driven approach is particularly challenging. Further studies that integrate current knowledge regarding gene expression profiling with clinical outcomes and robust data sets are required to fully understand the determinants of response to neoadjuvant treatment in oesophageal cancer and develop robust biomarkers for use in clinical practice. It must be noted that designing and implementing such trials are costly and resource-intensive. Ultimately, this need is best served using well-designed prospective, collaborative trials that utilize expertise at a range of institutions, increase patient numbers through multicentre recruitment, and use standardized approaches to reduce potential bias. One such example is the OCCAMS network—a UK-wide multicentre initiative that leverages the world-leading clinical, genomics, and bioinformatics expertise alongside industry partners and represents a significant opportunity to prospectively integrate genomic and transcriptomic data with stratified, adaptive clinical trials [113].

In conclusion, we have endeavoured to outline the current status of predictive transcriptomic biomarker development in neoadjuvant therapy in oesophageal cancer. The ability to stratify patients for neoadjuvant treatment in oesophageal cancer could dramatically improve outcomes in this poor-prognosis disease. At present, a greater understanding is needed regarding how the aforementioned molecular subtypes interrelate, the molecular determinants of response, and the major biological pathways involved. The wealth of genomic and transcriptomic data provided by national and international translational science and clinical trial collaborations in the coming years will provide unique opportunities to stratify patients for neoadjuvant therapy. Only by paying close attention to the issues of biomarker development can we address the challenge of delivering clinical impact in oesophageal cancer through the application of precision oncology.

## Supplementary data

Supplementary data is available at *Gastroenterology Report* online

## Authors’ contributions

A.L. and R.T. drafted the manuscript. All authors read and approved the final manuscript.

## Funding

This work was performed within the Irish Clinical Academic Training (ICAT) Programme. This work was supported by the Wellcome Trust and the Health Research Board [Grant Number 203930/B/16/Z], the Health Service Executive National Doctors Training and Planning, and the Health and Social Care Research and Development Division, Northern Ireland.

## Conflicts of interest

None declared.

## Supplementary Material

goaa065_Supplementary_Data
