# Supplementary material for: Transcriptomic biomarkers for predicting response to neoadjuvant treatment in oesophageal cancer
Source: Gastroenterol Rep (Oxf). 2021 Jan 8;8(6):411–24. doi: 10.1093/gastro/goaa065 (PMC7793050; doi:10.1093/gastro/goaa065)
Supplement: goaa065_Supplementary_Data [file goaa065_supplementary_data.docx]

| **Supplementary Table 1. Studies reporting mRNAs associated with response to neoadjuvant therapy in OAC and OSCC** | | | | | | | | | | | | | | | |
| --- | --- | --- | --- | --- | --- | --- | --- | --- | --- | --- | --- | --- | --- | --- | --- |
| **Study characteristics** | | | | | **Response**  **Responders vs Non-responders** | | | | | | | | **Survival** | | |
|  |  |  |  |  |  |  |  |  |  |  |  |  | **OS** | **RFS** | |
| **Author** | **Pathology (cases)** | **Signature** | **Responder vs non-responder** | **Cut-off/**  **fold change** | **Sensitivity** | **Specificity** | **Accuracy** | **PPV** | **NPV** | **AUC  (95% CI)** | **OR/HR  (95% CI)** | ***P* value (response)** | **HR**  **(95% CI),**  ***P* value** | | **HR**  **(95% CI),**  ***P* value** |
| Turkington  [47] | OAC | DDIR signature  (44 genes) | * | >2 fold | - | - | - | - | - | - | - | 0.033 | HR = 0.52 (0.31–0.88)  *P* = 0.015 | | HR = 0.61 (0.38–0.98) *P* = 0.042 |
| MacGregor  [60] | OAC | 7 DNA repair genes | ↓ | * | - | - | - | - | - | - | - | < 0.05 | ^a^ | | ^a^ |
| McLaren  [48] | OAC | *CCL28* | ↑ | - | - | - | - | - | - | - | LogHR = 1.53 (0.46–2.59) | 0.005 | - | | - |
|  |  | *DKK3* | ↓ | - |  |  |  |  |  |  | LogHR = -1.03  (-1.97, -0.10) | 0.031 |  |  |  |
| Rao  [51] | OAC | 113 genes | * | 1.4 fold | - | - | - | - | - | - | - | <0.05 | - | | - |
| Schauer  [49] | OAC | 86 genes | * | ≥2 fold | - | - | - | - | - | - | - | - | - | | - |
| Luthra  [59] | OAC | *IVL* | ↑ | - | - | - | - | - | - | - | - | 0.05 | - | | - |
| Fujishima  [67] | OSCC | 17 molecules | * | * | - | - | - | - | - | - | - | <0.05 | - | | - |
| Wen  [68] | OSCC | *LIMCH1 MMP1 C1orf226* | ↓ ↑ ↓ | >2 fold | T 82% V 54% | T 88% V 100% | T 86% V 81% | - | - | T: 0.97  (0.92–1) V: 0.82  (0.66–0.98) | T: OR = 15.97 (1.73–147.55) V: OR = 1.47 (1.07–2.01) | T 0.015 V 0.017 | - | | - |
| Motoori  [52] | OSCC | 199 genes | * | * | T 68% V 75% | T 93% V 83% | T 82% V 80% | T 88% V 75% | T 79% V 83% | - | - | <0.001 | - | | - |
| Pühringer-Oppermann  [69] | OSCC | *SMAD4* | ↑ | - | - | - | - | - | - | - | - | 0.032 | *P* > 0.05 | | - |
| Bollschweiler  [71] | OAC (56) OSCC (29) | 3 genes: *ERCC1  DPYD ERBB2* + ERCC1-SNP | ↓ ↓ ↓ | * | 70% | 90% | 80% | 89% | 74% | - | - | <0.05 | - | | - |
| Warnecke-Eberz  [72] | OAC (17) OSCC (24) | 17 genes (ANN analysis) | * | * | 80% | 91% | 85% | - | - | - | - | - | - | | - |
| Metzger  [73] | OAC (26) OSCC (40) | *STK11*; OAC only | ↑ | - | - | - | - | - | - | - | - | 0.048 | *STK11*  *P* = 0.022 | | - |
|  |  | *CUL2*: OAC + OSCC | ↑ | 1.7 |  |  |  |  |  |  |  | 0.02 | *CUL2*:  *P* = 0.032 | |  |
| Maher  [63] | OAC (10) OSCC (3) | 12 genes | * | >2 fold | T - V 100%^b^ | T - V 91% | T - V 95% | - | - | - | - | <0.01 | - | | - |
| Duong  [74] | OAC (25) OSCC (21) | 32 genes (OSCC only) | * | - | 100% | 67% | 76% | 55% | 100% | - | - | - | - | | - |
| Luthra  [61] | OAC (16) OSCC (2) ASCC (1) | 3 genes: *PERP S100A2 SPRR3* | ↑ ↑ ↑ | >2 fold | 86% | 85% | 85% | 75% | 92% | - | - | - | - | | - |

**Supplementary Table 1:**

**Legend:**

* = Individual genes not shown

- = Not reported

^a^ = Another gene combination predicts for survival

^b^ = 5 genes, in 74% of cases for whom a prediction could be made

↑ / ↓ = increased or decreased expression in responders compared with non-responders

**Abbreviations**: ANN, artificial neuronal network; AUC, area under curve; CI, confidence interval; DDIR, DNA damage immune response; HR, hazard ratio; NPV, negative predictive value; OAC, oesophageal adenocarcinoma; OR, odds ratio; OSCC, oesophageal squamous cell carcinoma; OS, overall survival; PPV, positive predictive value; RFS, relapse-free survival; T, training cohort; V, validation cohort.

| **Supplementary Table 2. Genes associated with predicting response to neoadjuvant treatment in mRNA studies, categorised according to the Hallmarks of Cancer** | | |
| --- | --- | --- |
| **Cancer hallmark** | **Number of genes** | **Percentage of total categorised genes (%)** |
| Sustaining proliferative signalling | 78 | 17.4 |
| Resisting cell death | 61 | 13.6 |
| Evading growth suppressors | 61 | 13.6 |
| Activating invasion and metastasis | 56 | 12.5 |
| Avoiding immune destruction | 47 | 10.5 |
| Tumour-promoting inflammation | 43 | 9.6 |
| Genome instability and mutation | 35 | 7.8 |
| Deregulating cellular energetics | 33 | 7.4 |
| Inducing angiogenesis | 22 | 4.9 |
| Enabling replicative immortality | 12 | 2.7 |
| Other - uncategorised | 59 |  |

.

| **Supplementary Table 3. Studies reporting miRNAs associated with response to neoadjuvant therapy in OAC and OSCC** | | | | | | | | | | | |
| --- | --- | --- | --- | --- | --- | --- | --- | --- | --- | --- | --- |
| **Study Characteristics** | | | **Response**  **Responders vs non-responders** | | | | | | | **Survival** | |
|  |  |  |  |  |  |  |  |  |  | **RFS** | **OS** |
| **Author** | **Pathology (cases)** | **Biomarker** | **Responder vs non-responder** | **Fold change** | ***P* value** | **Sensitivity (95% CI)** | **Specificity (95% CI)** | **Accuracy** | **AUC**  **(95% CI)** | **HR for RFS (95% CI),  *P* value** | **OR for OS,  *P* value** |
| **Chiam**  [91] | OAC | miR-4521/miR-340-5p | ↑ | - | 0.0002 | 95%  (86%–100%) | 89%  (67%–100%) | - | - | 7.5 (3.3–16.7) *P* = 0.015 | - |
|  |  | miR101-3p/miR-451a | ↑ | - | <0.0001 | 91% (77%–100%) | 89%  (67%–100%) | - | - | 21.3 (6.0–75.8) *P* = 0.011 | - |
|  |  | miR143-3p/miR-451a | ↑ | - | 0.002 | 91%  (77%–100%) | 89%  (67%–100%) | - | - | - | - |
| **Lynam Lennon**  [92] | OAC | miR-187 | ↑ | - | <0.01 | - | - | - | - | - | - |
| **Bibby**  [93] | OAC | miR-330-5p | ↑ | - | <0.01 | - | - | - | - | - | - |
| **Skinner**  [90] | OAC | miR-99b | ↓ | - | T: 0.016 V: 0.023 | - | - | - | - | - | - |
|  |  | miR-145* | ↓ | - | T: 0.009 V: 0.055 | - | - | - | - | - | - |
|  |  | miR-451 | ↓ | - | T: 0.009 V: 0.045 | - | - | - | - | - | - |
|  |  | miR-505* | ↓ | - | T: 0.05 V: 0.0013 | - | - | - | - | - | - |
|  |  | Combination |  |  | T: 0.008 V: 0.025 | - | - | - | T: 0.78  (0.52–0.98) V: 0.71  (0.51–0.84) | - | - |
| **Slotta-Huspenina**  [99] | OSCC | miR-194* | ↓ | >2 fold | <0.001 | - | - | - | 0.811  (0.694–0.927) | - | *P* = 0.052 |
|  |  | miR-665 | ↓ |  | 0.006 | - | - | - | 0.817  (0.704–0.930) | - | *P* = 0.197 |
|  |  | Combination |  |  |  | - | - | - | 0.824  (0.713–0.935) | - | - |
| **Wen**  [98] | OSCC | miR-145-5p | ↓ | >1.5 fold | - | - | - | - | - | - | - |
|  |  | miR-152 | ↓ |  |  |  |  |  |  |  |  |
|  |  | miR-193b-3p | ↑ |  |  |  |  |  |  |  |  |
|  |  | miR-376a-3p | ↓ |  |  |  |  |  |  |  |  |
|  |  | Combination |  |  | T: <0.001 V: <0.001 | T: 100% V: 83% | T: 100% V: 88% | T: 100% V: 87% | T: 0.927 (0.724–1.000) V: 0.868  (0.771–0.965) |  |  |
| **Sugimura**  [97] | OSCC | let-7b | ↑ | - | 0.014 | - | - | - | - | - | *P* = 0.128 |
|  |  | let-7c | ↑ | - | 0.032 | - | - | - | - | - | *P* = 0.032 |
| **Odenthal**  [100] | OAC (48) OSCC (40) | miR-192 | ↑ | - | 0.005^a^ | - | - | - | - | - | - |
|  |  | miR-194 | ↑ | - | 0.04^a^ | - | - | - | - | - | - |
| **Ko**  [101] | OAC (20) OSCC (5) | miR-296 | ↓ | 2.5 fold | 0.007 | - | - | - | - | - | - |
|  |  | miR-141 | ↑ | 2 fold | 0.019 |  |  |  |  |  |  |
|  |  | miR-31 | ↑ | 2 fold | 0.018 |  |  |  |  |  |  |
|  |  | HS-240 | ↓ | 3.2 fold | 0.04 |  |  |  |  |  |  |
|  |  | HS_217 | ↑ | 2 fold | 0.048 |  |  |  |  |  |  |
| **Lynam Lennon**  [102] | OAC (33) OSCC (4) | miR-31 | ↑ | - | 0.0279 | - | - | - | - | - | *P* > 0.05 |

**Supplementary Table 3:**

**Legend**:

- = Not reported

^a^ = OSCC only

↑ / ↓ = increased or decreased expression in responders compared with non-responders

**Abbreviations**: AUC, area under curve; CI, confidence interval; HR, hazard ratio; miRNA, microRNA; OAC, oesophageal adenocarcinoma; OR, odds ratio; OSCC, oesophageal squamous cell carcinoma; OS, overall survival; RFS, relapse-free survival; T, training cohort; V, validation cohort.
